# Hodgkin's Lymphoma: A Review of Neurologic Complications

**DOI:** 10.1155/2011/624578

**Published:** 2010-10-11

**Authors:** Sean Grimm, Marc Chamberlain

**Affiliations:** ^1^Northwestern University, Chicago, IL 60208, USA; ^2^Department of Neurology and Neurological Surgery, Seattle Cancer Care Alliance, Fred Hutchinson Cancer Research Center, University of Washington, 825 Eastlake Avenue E, P.O. Box 19023, MS-G4940, Seattle, WA 98109-1023, USA

## Abstract

Hodgkin's lymphoma is a hematolymphoid neoplasm, primarily of B cell lineage, that has unique histologic, immunophenotypic, and clinical features. Neurologic complications of Hodgkin's Lymphoma can be separated into those that result directly from the disease, indirectly from the disease, or from its treatment. Direct neurologic dysfunction from Hodgkin's Lymphoma results from metastatic intracranial spinal disease, epidural metastases causing spinal cord/cauda equina compression, leptomeningeal metastases, or intradural intramedullary spinal cord metastases. Indirect neurologic dysfunction may be caused by paraneoplastic disorders (such as paraneoplastic cerebellar degeneration or limbic encephalitis) and primary angiitis of the central nervous system. Hodgkin's lymphoma treatment typically includes chemotherapy or radiotherapy with potential treatment-related complications affecting the nervous system. Neurologic complications resulting from mantle-field radiotherapy include the “dropped head syndrome,” acute brachial plexopathy, and transient ischemic attacks/cerebral infarcts. Chemotherapy for Hodgkin's lymphoma may cause cerebral infarction (due to emboli from anthracycline-induced cardiomyopathy) and peripheral neuropathy.

## 1. Introduction

Hodgkin's lymphoma (HL) (previously termed Hodgkin's disease) is a hematolymphoid neoplasm, primarily of B cell linage, that has unique histologic, immunophenotypic, and clinical features. Its incidence is estimated to be 7400 cases per year in the United States [1]. The age distribution is bimodal; the first peak occurs between the ages of 15 and 30 years and the second in the sixth decade. Staging of the disease is classified according to the Cotswold scheme [2]. Treatment is based on disease stage and prognostic factors independent of stage such as bulky disease [1]. Treatment is usually multiagent chemotherapy, most often utilizing doxorubicin, bleomycin, vinblastine, and dacarbazine, radiotherapy (RT), or both.

Although neurologic complications from HL or its treatment are rare, there are several unique disorders that may be seen and are clinically recognizable (Algorithm 1). The neurologic disorders related to HL can be separated into those that result directly from HL, that is, metastatic complications, indirectly from HL, or from HL-directed treatment. Treatment of these various disorders is not unfortunately standardized due to the rarity of HL-related neurologic disorders, and consequently, management of these disorders reflects treatment for similar disorders occurring in other more common cancers.

## 2. Direct Neurologic Complications of HL

HL is predominantly a disease of the lymph nodes although extranodal sites of disease may be present in 10% of cases. Direct neurologic dysfunction results from intracranial metastases, metastases to the epidural space of the spinal cord with resultant spinal cord or nerve root compression, metastatic leptomeningeal disease, and intramedullary spinal cord metastases. In a retrospective review of HL, Wood and coltman reported intracranial metastases in 3% of patients and epidural spinal cord metastases in 3% of 353 patients with HL [3]. Other series have estimated central nervous system (CNS) involvement in HL less often (0.5%) likely reflecting improvement in systemic therapy with a corresponding decrease in metastatic disease [4]. 

### 2.1. Intracranial HL

Metastatic intracranial HL involvement is rare but well characterized [4]. The majority of cases are in patients with advanced, refractory disease, although intracranial disease may occasionally be present at HL diagnosis [5–7]. In literature review which included 36 patients with intracranial HL, the sites of involvement were brain parenchyma in 64% (cerebrum 44% and cerebellum/brainstem 20%), dura or leptomeninges in 19%, corpus callosum in 3%, pituitary in 3%, and unknown/not stated in 12% [7]. The presenting signs and symptoms reflect location of metastases and include cranial nerve palsies (55%), headaches (36%), weakness (33%), papilledema (19%), nausea and vomiting (17%), memory problems (17%), seizures (14%), gait disturbance (5%), and other (3%). Due to its rarity, treatment for intracranial metastatic HL is not well established but most often employs fractionated radiotherapy.

### 2.2. Epidural HL

Metastases of HL to the spinal epidural space may cause spinal cord or cauda equina compression [8–12]. The incidence of HL causing spinal cord compression has been estimated to be 0.2% [9]. Symptoms of spinal cord compression (not unique to HL) include back pain (progressive, worse when lying flat, and improved with walking), weakness, sensory loss, autonomic dysfunction (painless urinary retention, fecal incontinence, and impotence), and ataxia. Signs of spinal cord compression result in sensory level, paraparesis, hyperreflexia, and presence of the Babinski response. Cauda equina compression is characterized by back pain, dermatomal sensory loss, and asymmetric paraparesis. Because HL is usually chemosensitive, surgical decompression may not be necessary and if employed, may delay the delivery of definitive chemotherapy. Surgery, customarily vertebrectomy, is clearly indicated however if the diagnosis is uncertain or if there is evidence of spinal instability. Chemotherapy alone or chemotherapy followed by radiotherapy is effective treatment in the majority of patients [8, 9, 12].

### 2.3. Leptomeningeal HL

Cerebrospinal fluid (CSF) dissemination (leptomeningeal metastases) of HL similar to neoplastic meningitis due to other cancers is characterized by multifocal signs and symptoms affecting the brain (headache, gait difficulty, cognitive dysfunction, and seizures), cranial nerves (diplopia, hearing loss, and facial weakness), and spine and exiting nerve roots (weakness, sensory loss, and autonomic dysfunction). HL leptomeningeal metastases is an infrequent cause of neoplastic meningitis though small cases series have been reported [13–16]. Magnetic resonance imaging (MRI) may display focal or diffuse contrast enhancement of the meninges, subarachnoid nodules or intradural exiting nerve (brain or spinal cord) contrast enhancement or enlargement. CSF studies often reveal an increased opening pressure, lymphocytic pleocytosis, increased protein, and/or hypoglycorrhachia. A unique feature of leptomeningeal HL is that it is often associated with a CSF eosinophilic pleocytosis [13, 17, 18]. The mechanism of CSF eosinophilia in HL is unclear as it does not appear to be secondary to absolute blood eosinophilia [13]. Other diseases associated with CSF eosinophilia include parasitic diseases (cysticercosis, trichinosis, and helminth infection), tuberculous meningitis, symptomatic neurosyphilis, subacute sclerosing panencephalitis, and Coxsackie virus infection [19]. Identifying Reed-Sternberg cells in the CSF is the definitive diagnostic test for ascertaining HL-related leptomeningeal metastases [14, 15]. No standard treatment exists for leptomeningeal HL, but similar to other causes of lymphomatous meningitis, intra-CSF chemotherapy may provide palliation in conjunction with involved-field radiotherapy [13, 20, 21].

### 2.4. Intramedullary Spinal Cord HL

The spine parenchyma (intramedullary spine) is a rare site of metastatic disease in patients with HL [22, 23]. Intramedullary spinal metastases arise from direct hematogenous spread to the parenchyma of the spinal cord or by centripetal growth of tumor along spinal nerve roots with secondary invasion of the spinal cord. Signs and symptoms are similar to those with epidural spinal cord compression, except back pain may not be prominent. Chemotherapy and/or radiation therapy are the preferred treatment options.

## 3. Indirect Neurologic Complications of HL

### 3.1. Paraneoplastic Syndromes

Paraneoplastic syndromes are disorders of tissue or organ function caused by a cancer but at a site remote from the cancer or its metastases [24]. Any portion of the nervous system (central or peripheral) can be affected by a paraneoplastic syndrome. Specificity of nervous system paraneoplastic disorders is reflected by involvement of one or several anatomic sites or by nervous system cell types affected. The majority of paraneoplastic disorders are thought to be immune-mediated [25]. Most patients with a paraneoplastic syndrome have antibodies in their serum or CSF that react with both the nervous system and the underlying cancer. Although there can be overlap, a specific antibody is usually associated with a specific clinical syndrome and a restricted subgroup of cancer.

Several paraneoplastic syndromes have been reported in patients with HL of which cerebellar degeneration is the best characterized. There is a strong association between the circulating antineuronal antibody anti-Tr with paraneoplastic cerebellar degeneration and HL [26]. Unlike other paraneoplastic syndromes in which the paraneoplastic syndrome precedes the diagnosis of cancer, the paraneoplastic disorder of HL begins after the diagnosis of HL or when the patient is in remission [26–28]. In one series of 21 patients, the majority were young (median age 44 years) men (18/21). Diagnosis of HL preceded neurologic symptoms by 1 to 54 months in 17/21 patients and during remission in four [27]. The typical presentation is pancerebellar dysfunction (dysarthria, nystagmus, truncal ataxia, and appendicular ataxia) developing over months. Plasmapheresis, corticosteroids, and immunosuppressant medications are of no apparent benefit. The syndrome may resolve spontaneously otherwise there appears to be no effective therapy [27, 28]. 

A separate, distinct paraneoplastic cerebellar degeneration syndrome associated with autoantibodies to a mouse metabotropic glutamate receptor (mGLuR1) in two patients with HL has been reported [29]. The patients presented with subacute cerebellar ataxia several years after successful treatment for HL. One patient experienced improvement of their ataxia after treatment with prednisone, intravenous immunoglobulin, and plasma exchange. A third case of mGLuR1 paraneoplastic cerebellar degeneration without history of HL has been described, raising the question whether the association with HL in the first series is coincidental [30]. 

Anti-NMDA (N-methyl-D-aspartate) receptor encephalitis is a paraneoplastic syndrome characterized by limbic encephalitis (memory loss, confusion, seizures, and psychiatric abnormalities), rhythmic movement disorder, hypoventilation requiring ventilation, and autonomic instability, mostly occurring in women with ovarian teratoma [31]. Zandi reported a patient who presented with anterograde memory impairment at HL relapse [32]. Antibodies to the NMDA receptor were identified in serum and CSF. The patient improved with aggressive immunotherapy. Several other cases of paraneoplastic limbic encephalitis without an identified autoantibody have been reported in association with HL [33, 34]. Because the patients presented prior to the recognition of anti-NMDA-receptor encephalitis, their NMDA antibody status is unknown.

Cases of acute inflammatory demyelinating polyradiculopathy (Guillain-Barre syndrome) occurring in patients undergoing active treatment for HL or at disease recurrence have been reported. Whether the neurologic syndrome was associated with HL as a paraneoplastic syndrome, secondary to an aspect of the treatment, or coincidental is not clear [35–38]. 

Other possible paraneoplastic complications of HL include chorea and ataxia, subacute sensory neuropathy, motor neuron disease, myasthenia gravis, stiff person syndrome, and brachial neuropathy [39–44].

### 3.2. Primary Angiitis of the Central Nervous System (PACNS)

PACNS is a noninfectious granulomatous angiitis that affects small arteries of the leptomeninges, and parenchyma of the brain and spinal cord in the absence of systemic vasculitis. Patients present with headache, encephalopathy, seizure, hemorrhage, and multifocal infarcts [45]. The etiology is unknown, although there may be an association between PACNS and HL. The diagnoses of PACNS and HL are often made simultaneously or closely correlated in time [46–49]. In addition to arterial involvement, recurrent cerebral venous thrombosis, despite anticoagulation in a patient with HL, has been described. The venous thrombosis was thought to be inflammatory in etiology since the patient had aseptic meningitis and experienced a dramatic response to rituximab and steroids [50]. Other conditions that have been associated with PACNS include herpes zoster, non-Hodgkin's lymphoma, human immunodeficiency virus, and Sjogren's syndrome [49]. Since herpes zoster infections are common in patients with HL [51], the relationship between zoster vasculitis and PACNS associated with HL is unclear.

## 4. Neurologic Complications of HL Treatment

### 4.1. Neurologic Complications of Radiotherapy for HL

Traditionally, standard therapy for HL included “mantle field” radiotherapy (RT). The mantle field encompasses the submandibular, cervical, supraclavicular, infraclavicular, axillary, mediastinal, subcarinal, and hilar lymph nodes. Concern regarding the long-term complications of RT has led to tailored treatment protocols in line with prognostic factors that allow for reductions in the amount and extent of RT [52]. 

#### 4.1.1. Dropped Head Syndrome

The dropped head syndrome is characterized by severe weakness of neck extensor muscles causing an inability to extend the neck which results in a posture with the head flexed forward and chin on chest deformity. An association of dropped head syndrome with mantle field RT was first reported by Johansson et al. [53]. Since then, the “dropped head syndrome” has been increasingly recognized as a long-term complication of high dose mantle field RT [54–56]. The classical presentation is severe weakness and atrophy of cervical and shoulder girdle musculature (splenius capitis, supraspinatus, infraspinatus, trapezius, sternocleidomastoid, and deltoid muscles) leading to the dropped head syndrome. The symptoms begin many years (most cases occuring >20 years) after high-dose mantle field radiotherapy. Sensory loss, cramps, paresthesias, fasciculations, and myokymia on EMG are not present. The weakness may progress over years but does not extend beyond the initially involved muscle groups. The pathophysiology of this rare RT complication is unclear. It is thought to result from a combination of primary muscle damage and upper cervical anterior horn/nerve root lesions. Both myopathic and neurogenic EMG changes have been reported. Muscle biopsy reveals noninflammatory myopathic changes, presence of nemaline rod bodies, or features of muscle denervation. Treatment is supportive often employing a cervical collar to maintain the head upright.

#### 4.1.2. Brachial Plexopathy

Acute brachial plexopathy has been reported in several HL patients during treatment with high dose mantle field RT [57, 58]. The patients developed painful, acute brachial plexus dysfunction (shoulder and arm pain followed by arm/hand sensory loss and lower motor neuron weakness) within days to weeks of starting RT; improvement occurred despite continued RT. Clinically, the syndrome is indistinguishable from acute idiopathic brachial neuritis. The pathogenesis is unknown, but an immune-mediated neuropathy precipitated by the radiotherapy has been postulated [57]. Late-delayed brachial plexus neuropathy following mantle RT has also been reported and like other cancer-related radiation plexopathies is painless and associated with myokymia [59].

#### 4.1.3. Episodic Neurologic Dysfunction

Feldmann and Posner described 14 patients (12 women and two men, 26 to 51 years of age) in remission and without evidence of HL who presented with episodic and presumed vascular neurologic dysfunction [60]. All but one received mantle field RT. Most patients experienced visual disturbances including attacks of “flashing lights” in one or both eyes, monocular visual loss, or hemianopia. Other episodic neurologic symptoms included language dysfunction, segmental motor, or segmental sensory defects. Absence of headache argued against a migrainous phenomenon. The authors speculated that these episodes represented transient ischemic attacks (TIA); only one patient was diagnosed with cerebral infarction at presentation or on followup. The disorder appears to be benign; nine became asymptomatic (3 spontaneously, 1 patient with carotid stenosis underwent endarterectomy, and 5 received antiplatelet therapy). Despite the seemingly benign nature of episodic neurologic phenomena in patients successfully treated for HL, cerebral infarction may be a late treatment complication [61]. Patients presenting with episodic neurologic symptoms should undergo complete neurologic workup including echocardiography to look for valvular heart disease and cardiomyopathy, cervical and aortic angiography, and coagulation studies.

#### 4.1.4. Neurologic Complications of Chemotherapy for HL

The standard chemotherapy regimen for HL includes doxorubicin, bleomycin, vinblastine, and dacarbazine (ABVD). Doxorubicin causes a cumulative, dose-dependent cardiomyopathy which can lead to transient ischemic attacks (TIA) or cerebral infarction from cardiac emboli [62]. Liposomal doxorubicin often causes plantar-palmar erythrodysesthesia (hand and foot syndrome) [63]. Bleomycin may cause cerebral or myocardial infarcts in patients who were successfully treated for HL [64]. Vinblastine may cause a predominantly length-dependent small fiber sensory peripheral neuropathy. The mechanism of neuropathy is thought to be interference of axonal transport due to tubulin binding. The major site of damage is thought to be the dorsal root ganglion which lies outside the blood-nerve barrier [65]. Dacarbazine in isolation rarely causes neurologic dysfunction.

Mechlorethamine, vincristine, procarbazine, and prednisone (MOPP) is another chemotherapy regimen that has been used for HL. Mechlorethamine is rarely neurotoxic, but encephalopathy and hearing loss have been reported after standard infusion [66, 67]. Vincristine is the most neurotoxic vinca alkaloid; it causes a dose limiting mixed motor-sensory neuropathy in the majority of patients [68]. The earliest complaint is tingling and paresthesias in the fingertips and later the toes [65]. Fine motor movements of the fingers and toes are often impaired. Loss of Achilles reflexes is the earliest finding on examination. With continued treatment, other deep tendon reflexes disappear. Objective sensory loss is uncommon early but with progression clinical findings becomes evident and involves the classic glove and stocking distribution. Patients often experience weakness of extensor muscles particularly in the feet. The symptoms usually develop a few weeks following initiation of therapy and progress for several weeks after drug discontinuance. Symptoms are usually reversible once treatment is discontinued if treatment is interrupted early and before clinical signs are manifest. Neurophysiologic studies are consistent with an axonal neuropathy. In addition to peripheral neuropathy, vincristine causes mononeuropathies involving peripheral or cranial nerves and autonomic dysfunction. Dysfunction of the oculomotor nerve, recurrent laryngeal nerve, facial nerve, acoustic nerve, and optic nerve have been described [65]. Autonomic dysfunction includes constipation, paralytic ileus, bladder atony, impotence, and postural hypotension. Procarbazine is rarely neurotoxic but may cause encephalopathy and reversible peripheral neuropathy [65].

## 5. Conclusions

Because HL affects both the central and peripheral nervous systems, HL may present in a pleomorphic manner and mimic a variety of neurologic syndromes. HL is associated with a myriad of neurological complications that occur both as a direct consequence of HL (intraparenchymal brain metastases, epidural spinal cord compression, HL meningitis, and dural metastases) and indirectly due to treatment or paraneoplastic disorders. The majority of nervous system complications of HL are metastatic or treatment related in etiology (Algorithm 1). Metastatic complications are treated in a manner similar to other cancers with comparable metastatic syndromes. Peripheral nervous system disorders are nearly always treatmentrelated (radiation-induced brachial plexopathy or dropped head syndrome and vinca alkaloid-induced peripheral neuropathy) and respond best to discontinuance of the neurotoxic agent and supportive care. Familiarity with the various neurologic disorders related to HL is critical to make the correct diagnosis and institute the appropriate treatment.

## Figures and Tables

**Algorithm 1:**
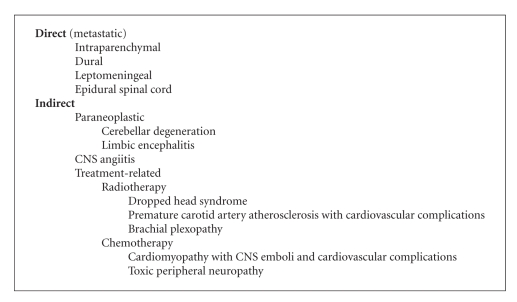
Neurological complications of Hodgkin's lymphoma.
